# Acetylation Disfavors Tau Phase Separation

**DOI:** 10.3390/ijms19051360

**Published:** 2018-05-04

**Authors:** Josephine C. Ferreon, Antrix Jain, Kyoung-Jae Choi, Phoebe S. Tsoi, Kevin R. MacKenzie, Sung Yun Jung, Allan Chris Ferreon

**Affiliations:** 1 Department of Pharmacology and Chemical Biology, Baylor College of Medicine, Houston, TX 77030, USA; Kyoungjae.Choi@bcm.edu (K.-J.C.); Phoebe.Tsoi@bcm.edu (P.S.T.); Kevin.MacKenzie@bcm.edu (K.R.M.); 2 Advanced Technology Cores, Baylor College of Medicine, Houston, TX 77030, USA; antrixj@bcm.edu; 3 Department of Pathology and Immunology, Baylor College of Medicine, Houston, TX 77030, USA; 4 Department of Biochemistry and Molecular Biology, Baylor College of Medicine, Houston, TX 77030, USA; syjung@bcm.edu

**Keywords:** intrinsically disordered protein, membrane-less organelle, neurodegenerative disease, p300 HAT acetylation, post-translational modification, protein aggregation, Tau fibrillation

## Abstract

Neuropathological aggregates of the intrinsically disordered microtubule-associated protein Tau are hallmarks of Alzheimer’s disease, with decades of research devoted to studying the protein’s aggregation properties both in vitro and in vivo. Recent demonstrations that Tau is capable of undergoing liquid-liquid phase separation (LLPS) reveal the possibility that protein-enriched phase separated compartments could serve as initiation sites for Tau aggregation, as shown for other amyloidogenic proteins, such as the Fused in Sarcoma protein (FUS) and TAR DNA-binding protein-43 (TDP-43). Although truncation, mutation, and hyperphosphorylation have been shown to enhance Tau LLPS and aggregation, the effect of hyperacetylation on Tau aggregation remains unclear. Here, we investigate how the acetylation of Tau affects its potential to undergo phase separation and aggregation. Our data show that the hyperacetylation of Tau by p300 histone acetyltransferase (HAT) disfavors LLPS, inhibits heparin-induced aggregation, and impedes access to LLPS-initiated microtubule assembly. We propose that Tau acetylation prevents the toxic effects of LLPS-dependent aggregation but, nevertheless, contributes to Tau loss-of-function pathology by inhibiting Tau LLPS-mediated microtubule assembly.

## 1. Introduction

Tau inclusions are key components of neurofibrillary tangles (NFTs) a recurring pathological feature for several neurodegenerative diseases including Alzheimer’s disease (AD) [1,2,3,4]. There are two prevailing hypotheses on the mechanism of Tau pathology linked to protein misfolding and aggregation, both of which are not mutually exclusive [5,6,7]. One is that Tau has intrinsic aggregation motifs that enable fibrillation, leading to gain-in-toxic function(s) [8,9,10,11] exacerbated by the inability of the cellular degradation machinery to remove misfolded or aggregated Tau [12,13]. Another pathological mechanism is that aggregation-promoting Tau accumulation stems from loss-of-normal function(s). Tau is essential for microtubule dynamics and stability; impairment of this function linked to Tau sequestration into aggregates results in neuronal loss [14]. Alterations in protein sequence and structure (such as truncations, mutations, or post-translational modifications) contribute to both Tau loss-of-normal function and gain-in-toxic dysfunction by affecting the protein’s ability to bind microtubules or propensity to misfold and aggregate [6,7,11].

Tau is an intrinsically disordered protein (IDP), a class of proteins characterized by a high degree of structural flexibility, conformational heterogeneity and binding promiscuity. Often, these properties not only allow for complex functions involving networks of interactions, but also facilitate dysfunctions as a result of misfolding or aggregation [15,16,17]. Tau is rich in serine/threonine (S/T) and lysine (K) residues, and is known to undergo post-translational modifications (PTMs), such as phosphorylation, acetylation, ubiquitination, and sumoylation. These PTMs are linked to both Tau function and pathology [18]. Phosphorylation is known to modulate Tau’s ability to promote microtubule assembly. Abnormal Tau hyperphosphorylation, however, results in fibrillation, as evidenced by hyperphosphorylated Tau being the primary component of NFTs [1]. At least 20 phosphorylation sites [14] and 23 acetylation sites [19,20,21] have been reported for Tau.

**Figure 1:**
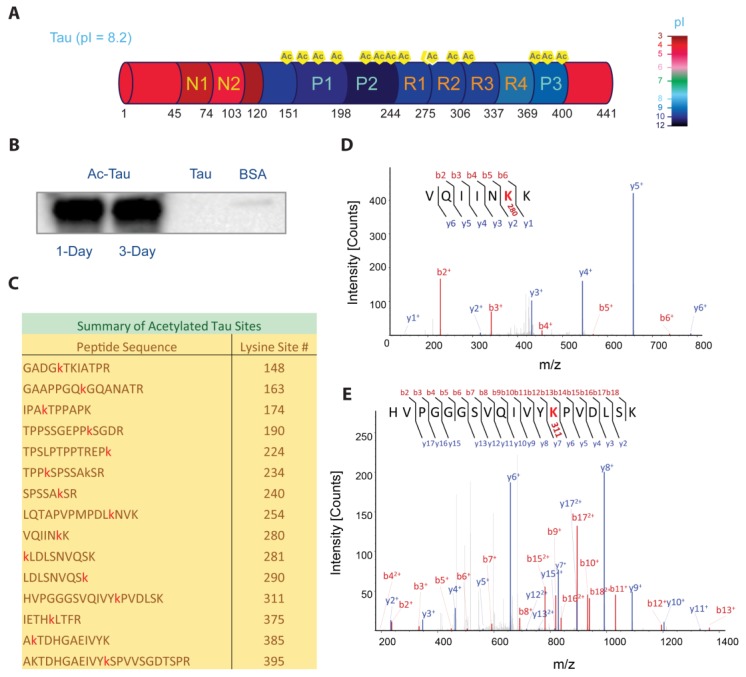
Tau hyperacetylation. (**A**) Domain organization of Tau. Protein segments are color-coded to reflect their respective pIs using the provided color palette. (**B**) In vitro Tau acetylation was verified by Western blot against acetyl-lysine (left to right lanes: acetylated Tau after one- and three-day reaction incubations, and negative controls using wild-type Tau and BSA, respectively). (**C**) Tau lysine acetylation sites (red) identified by mass spectrometry. (**D**,**E**) MS/MS spectra of peptides that contain the hexapeptide aggregation motifs VQIINK_280_K_281_ and VQIVYK_311_, showing acetylation at K280 and K311, respectively (shown in red). The ‘b’ ions (shown in red) represent fragment peaks generated from the amino to carboxyl terminus. The ‘y’ ions (shown in blue) represent fragment peaks generated from the carboxyl to amino terminus. The suffix numbers represent the corresponding number of amino acids. See Section 3 for details.

Tau is a macromolecular polyampholyte consisting of negatively-charged N- and C-terminal domains, and a positively-charged central Proline-rich (P) domain with microtubule binding regions (MTBR, R1–R4; Figure 1A). An increase in negative charge via phosphorylation or removal of lysine positive charge by acetylation can have significant effects on Tau function (microtubule assembly/stabilization) and dysfunction (Tau aggregation). Tau acetylation can be mediated by p300/CREB-binding protein (CBP) HAT [19,20,21,22,23], and reports indicate that Tau, itself, has intrinsic acetyltransferase activity [19,20,22]. In fact, hyperacetylated Tau (Ac-Tau) has been used as a diagnostic marker for AD [20,21,24]. Although the role of hyperphosphorylation in facilitating Tau pathological aggregation is not debated, there are conflicting reports on the role of hyperacetylation in Tau pathology. Several groups have found acetylated Tau in pathological inclusions in vivo [20,21], as well as in co-deposits with hyperphosphorylated Tau [21]. However, Cook et al. report that acetylation at key Tau motifs (K_259/353_IGS) can be protective through the inhibition of phosphorylation of a nearby serine that otherwise would promote aggregation [23]. In addition, observations from in vitro experiments are contradictory: Cohen et al. report that acetylation accelerates Tau heparin-induced fibrillation [20], whereas others indicate that acetylation inhibits Tau filament assembly [19,23].

Liquid-liquid phase separation (LLPS) has recently gained attention as a physical mechanism for proteins to self-assemble into compartments termed membrane-less organelles [25,26,27,28]. The LLPS-mediated enrichment of proteins into membrane-less organelles, such as stress granules [29], provides “hotbeds” or seeds for protein aggregation [30,31,32]. LLPS was shown to initiate the aggregation of several neurodegenerative disease-associated proteins, such as FUS [30], TDP-43 [31,33], and hnRNPA1 [34]. Recently, Tau LLPS has been implicated in both the functional role of Tau in promoting microtubule assembly [35] and dysfunction in initiating Tau self-interaction and fibrillation [36,37,38]. Although it has been shown that hyperphosphorylation accelerates Tau LLPS and aggregation, consistent with hyperphosphorylated Tau’s abundance in pathological inclusions [36,37], there are no reports on the role of acetylation on Tau phase separation and LLPS-mediated aggregation. Since Tau LLPS is expected to be strongly influenced by electrostatics, here, we investigate the role of acetylation in driving LLPS and determine if this role is consistent with the current hypothesis that LLPS can initiate and mediate Tau aggregation.

## 2. Results and Discussion

### 2.1. p300-Mediated Acetylation of Tau

Hyperacetylated Tau (Ac-Tau) was prepared from wt Tau using p300 HAT (see Section 3). Tau acetylation was verified by Western blot against acetyl-lysines (Figure 1B) and mass spectrometry (Figure 1C–E). We identified 15 acetylation sites (99% sequence coverage) using tandem mass spectrometry (Figure 1C; Section 3), including K148 near the Tau N-terminal domain; K163, K174, K190, K224, K234, and K240 in the P1–2 regions; K254, K280, K281, K290, and K311 in the microtubule binding region (MTBR, R1–R4); and, K375, K385, and K395 in the P3 region (Figure 1A,C). Figure 1D,E shows the representative MS/MS spectra (VQIINK_280_K_281_ and VQIVYK_311_, respectively). Fragmented b (red) and y (blue) ions from low energy collisions in mass spectrometer are marked. The b and y ions refer to the peaks corresponding to the prefix ions observed sequentially in the spectrum with each prefix offset from the previous by the mass of an amino acid. A 42.016 Da increase in mass difference due to lysine acetylation is included in the sequential mass difference to construct the peptide sequence. The acetylation sites that were identified are consistent with previous reports [19,20,21]. Notably, K280/K281 and K311, which are in the hexapeptide aggregation motifs VQIINK_280_K_281_ and VQIVYK_311_, were found to be acetylated (Figure 1D,E). These motifs play critical roles in Tau interaction with negatively-charged microtubules and with polyanions such as heparin [9]. If we assume complete acetylation, we expect the theoretical pI for full-length Tau to change from 8.2 to 5.5 (Prot pi web tool; https://www.protpi.ch). Such modifications can significantly alter Tau electrostatic properties, with 50% acetylation already corresponding to a pI of 6.2. Interestingly, the observed sites of acetylation are concentrated in the positively-charged central region of Tau (Figure 1A).

### 2.2. Acetylation Changes Tau Phase Behavior

**Figure 2:**
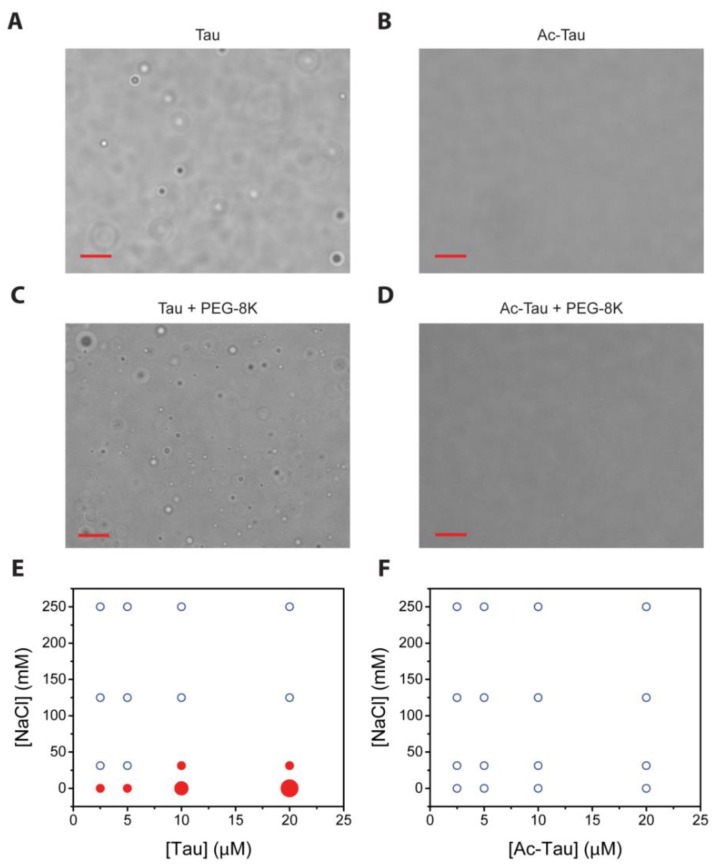
Acetylation disfavors Tau liquid-liquid phase separation (LLPS). Droplet formation of wt Tau versus hyperacetylated (Ac-Tau), respectively, in the absence (**A**,**B**) and presence of the crowding agent PEG-8K (**C**,**D**). Phase transition maps of Tau versus Ac-Tau (in the absence of crowders), respectively (**E**,**F**). The diagrams present protein and salt concentration dependencies of droplet formation. Blue open circles represent conditions of minimal droplet formation (<5 droplets/frame); small red solid circles represent 5–20 droplets, medium red solid circle 20–100 droplets, and large red circle >100 droplets. The bars in (**A**–**D**) represent 10 µm. See Section 3 for details.

LLPS has recently been observed for wt and hyperphosphorylated Tau, and truncation mutant (K18) [35,36,37,38]. At low salt conditions (5 mM sodium phosphate, pH 7.8), we observed near-instantaneous formation of wt Tau droplets (Figure 2A). Subsequent fusions indicate the liquid nature of the wt Tau droplets. We characterized the protein concentration (2.5–20 µM) and salt concentration (0–250 mM NaCl) dependencies of wt Tau LLPS. Consistent with the literature [37,38], higher salt concentrations disfavor LLPS and higher protein concentrations favor LLPS (Figure 2E). For the case of Ac-Tau, we observed a dramatic reduction in droplet formation (Figure 2B,F). Similar results were also observed when LLPS experiments were performed with wt Tau or Ac-Tau in the presence of a crowding agent (10% PEG 8K, 200 mM NaCl, 10 mM acetate, 10 mM glycine, 10 mM sodium phosphate, pH 7.5; Figure 2C,D). Thus, independent of the presence or absence of crowding, the hyperacetylation of Tau disfavors LLPS.

Interestingly, even though both hyperphosphorylation and hyperacetylation decrease the overall pI of Tau, the two PTMs seem to have opposite effects on LLPS. In contrast to LLPS enhancement by hyperphosphorylation [36,37], hyperacetylation clearly disfavors Tau LLPS (Figure 2). Further experiments performed in identical or comparable conditions using the same Tau constructs, full-length or otherwise, are needed for a clear and direct comparison of LLPS behaviors of hyperphosphorylated, hyperacetylated, and wt Tau proteins. Nevertheless, we think that hyperacetylation disfavors full-length Tau LLPS by neutralizing the lysine positive charges, thereby affecting opposite-charge attractions that help support Tau self- and mesoscale interactions. Our data also give direct support that electrostatics plays a major role in Tau LLPS.

### 2.3. Acetylation of Tau Inhibits Heparin-Induced Aggregation

**Figure 3:**
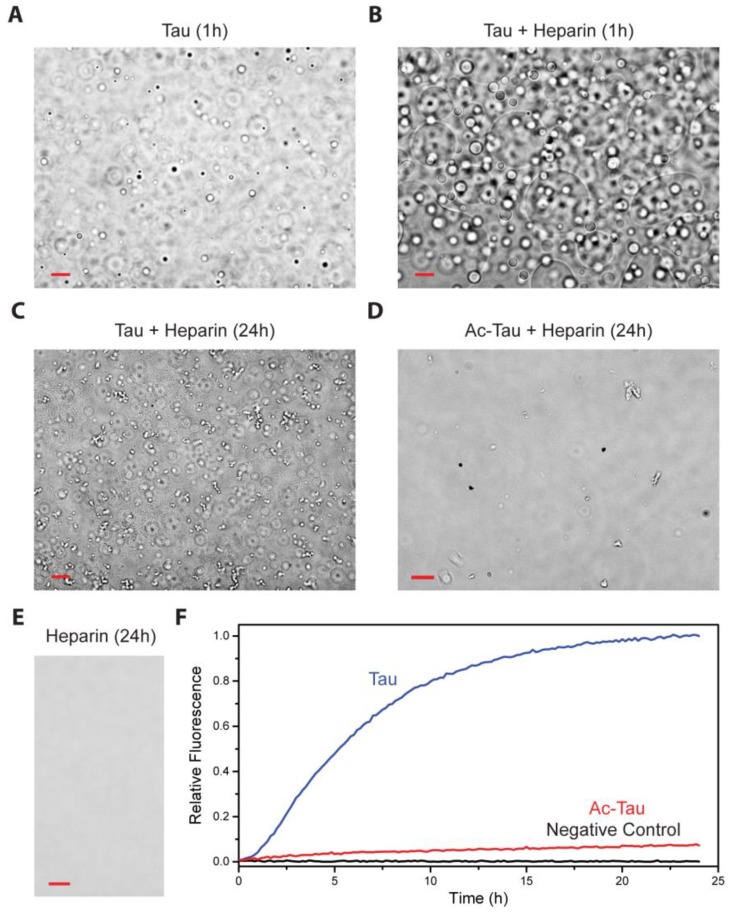
Acetylation disfavors heparin-induced Tau aggregation. (**A**,**B**) Heparin accelerates non-acetylated wt Tau LLPS. The presence of heparin (5 µM heparin: 20 µM Tau ratio) results in more droplets and larger fused droplets at the bottom of the dish. Droplet formation was not observed for Ac-Tau in the absence (Figure 2B,D) or presence of heparin (24 h, (**D**)). After 24 h incubation, an abundance of irregularly-shaped oligomers/aggregates was observed for wt Tau but not for Ac-Tau (**C**,**D**). No aggregation/LLPS was observed for the heparin control (**E**). Ac-Tau displayed minimal aggregation compared to wt Tau as reported by Th T fluorescence assay (**F**). Scale bars represent 10 µm. See Section 3 for details.

Heparin has been widely used to induce and accelerate Tau aggregation [11]. Utilizing a truncated Tau construct, Ambadipudi et al. demonstrated that heparin promotes Tau fibrillation via LLPS [37]. Similarly, we observed that heparin induces LLPS of full-length wt Tau and facilitates subsequent protein aggregation (Figure 3A–C,F). In contrast, Ac-Tau failed to undergo heparin-induced LLPS in the same experimental conditions (Figure 3D). Additionally, Ac-Tau (relative to wt Tau) exhibited a dramatic decrease in the fibrillation rate as reported by Th T fluorescence (Figure 3F). Residues in the VYINK_280_K_281_ and VQIVK_311_ regions of the Tau microtubule binding repeats (R1–R4), which we identified as Tau acetylation sites (Figure 1), are also known interaction sites for heparin [39]. Thus, the observed effects of acetylation on Tau heparin-induced aggregation can be attributed to the loss of binding to heparin.

Although heparin accelerates wt Tau LLPS, it is unknown whether heparin is equally distributed in the Tau-rich and Tau-poor phases (which we think to be unlikely). LLPS, nevertheless, allows Tau to co-localize and thereby concentrate, with the Tau-rich condensed phase facilitating Tau aggregation nucleation and/or seeding.

### 2.4. Acetylation of Tau Prevents Access to LLPS-Mediated Microtubule Assembly

**Figure 4:**
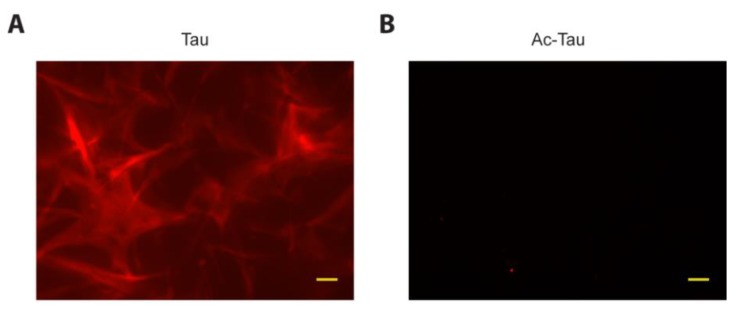
Tau acetylation prevents access to LLPS-mediated microtubule assembly. (**A**,**B**) Fluorescence microscopy images showing microtubule assembly from mixtures of rhodamine-labeled and unlabeled tubulin heterodimers with Tau or Ac-Tau, respectively. Scale bars represent 10 µm. See Section 3 for details.

A recent report by Hernandez-Vega et al. suggests that Tau phase separated droplets (induced using the crowding agents PEG, Ficoll or dextran) can initiate microtubule assembly [35]. To assess LLPS-mediated microtubule assembly by wt Tau and Ac-Tau independent of crowding agents, we performed our phase separation experiments in low-salt conditions. After mixing rhodamine-labeled and unlabeled tubulin heterodimers with wt Tau, we observed an initial increase in solution turbidity. The ensuing dynamic microtubule assembly was visible by fluorescence microscopy within 1 h of incubation (Figure 4A). In contrast, Ac-Tau neither displayed turbidity nor detectable microtubule assembly up to 18 h of incubation (Figure 4B). Whereas previous studies have shown that acetylation reduces Tau’s ability to bind to microtubules [20], our data clearly demonstrates that the failure of Ac-Tau to undergo LLPS affects its potential for microtubule assembly.

Our in vitro data indicate that Ac-Tau is less prone to aggregation as compared to wt Tau. Cryo-EM structures of AD patient-derived filaments indicate that Tau residues 306–378 form the amyloid core [40]. The stable core is composed of several β-strands that pack intra- and inter-molecularly, with β1 (_306_VYINK_311_) in close proximity to β8 [40]. Our results show that in Ac-Tau, K311 (β1), and K375 (β8) are both acetylated; we speculate that this influences interactions within the amyloid core, and contributes to inhibition of Tau aggregation. Further experiments on the acetylation of the amyloid core residues will be needed to directly assess the effect of Tau acetylation on the amyloid structure.

Recent reports suggest that Tau aggregation is accelerated through LLPS [36,37]. Our data clearly show that acetylation decreases or abolishes Tau LLPS. Our findings are consistent with an LLPS-mediated model of aggregation (but do not prove whether such a mechanism is operative in vivo). Since acetylation reduces the propensity of Tau to undergo LLPS, we conclude that acetylation in vivo is unlikely to enhance or lead directly to condensation-mediated aggregation, in contrast to the demonstrated effect of hyperphosphorylation [36]. It is, however, possible that combinations of phosphorylations and acetylations can favor LLPS and/or aggregation; future experiments with Tau bearing homogeneous PTMs will be needed to address this conclusively.

**Figure 5:**
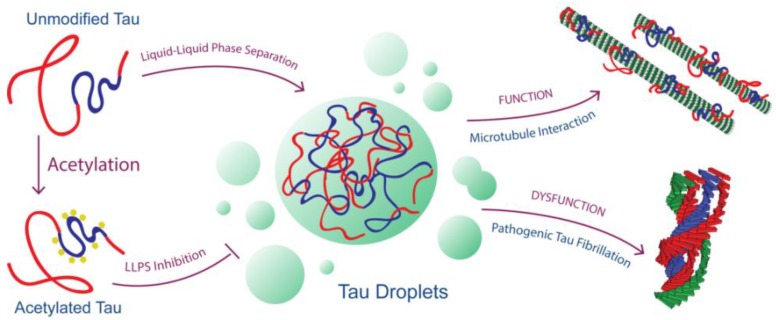
Model for Tau’s loss of physiologic function and gain of pathologic dysfunction linked to its ability to undergo LLPS as modulated by acetylation.

Tau participates in microtubule formation and stabilization, and Tau LLPS has been shown as a mechanism by which a Tau-rich condensed phase can recruit tubulin dimers and facilitate their assembly [20]. Acetylation at key Tau sites that interfere with tubulin binding would affect this function, as would acetylation that disfavors partitioning of Tau into a Tau-rich phase. Thus, we speculate that the primary contribution of Tau acetylation to cellular dysfunction is not through a gain-of-function mechanism, such as toxic aggregation, but through a loss of physiologic function mechanism (i.e., reduced binding to tubulins/microtubules, and decreased LLPS-mediated initiation of microtubule assembly; Figure 5).

Less direct effects on physiologic Tau function may also be important. Many of the same lysines (K254, K311, and K353) implicated as sites of ubiquitination [41] are also sites of acetylation and, thus, might be involved in evading the ubiquitin-lysosome proteasomal degradation machinery. Acetylation has been shown to inhibit Tau degradation by inhibiting its ubiquitination [21], and results in the accumulation of Tau, including hyperphosphorylated Tau. The presence of lysine deacetylase (SIRT1) has been shown to inhibit neuronal loss in an AD mouse model and deletion of SIRT1 results to pathologic levels of Tau in vivo [42]. Cross-talk between the different PTMs has also been reported. For example, hypoacetylation of Tau at key KIGS motifs in the *R1*-*4* regions increases vulnerability to hyperphosphorylation, which leads to filament aggregation [23]. Hyperphosphorylation of Tau has been reported to enhance Tau LLPS. However, other reports also show that hyperphosphorylation reduces microtubule assembly [14]. Thus, LLPS-mediated mechanisms by hyperphosphorylated Tau could be detrimental for both function and dysfunction pathways (Figure 5). We plan to carry out further experiments on hyperphosphorylated Tau to assess how this PTM of Tau can modulate microtubule assembly and protein aggregation, both in LLPS and non-LLPS conditions. Nevertheless, we speculate that the hyperacetylation of Tau is detrimental to Tau function, but not instrumental to LLPS-mediated Tau dysfunction (Figure 5). It would also be interesting to know the cross-talks between hyperphosphorylation and hyperacetylation in LLPS-mediated microtubule assembly and promotion of pathologic fibrils. Can hyperphosphorylated Tau also recruit hyperacetylated Tau into droplets? If so, this might explain the presence of hyperacetylated Tau in pathological inclusions of hyperphosphorylated Tau.

In conclusion, our data affirm the importance of electrostatics in Tau LLPS. Furthermore, we show that hyperacetylation disfavors Tau LLPS and, as a consequence, LLPS-facilitated aggregation. Finally, by preventing access to LLPS-mediated microtubule assembly and stabilization, hyperacetylation contributes to Tau dysfunction primarily through a loss-of-function mechanism.

## 3. Materials and Methods

### 3.1. Tau Expression and Purification

Wild-type (wt) Tau (2N4R isoform; 441 residues) plasmid (Addgene plasmid #16316, a gift from Peter Klein) was transformed into *Escherichia coli* BL21 star cells. Cells were grown at 37 °C in Terrific Broth medium in the presence of kanamycin until the optical density at 600 nm (OD_600_) reaches 0.8–1.0, then induced with 1 mM isopropyl β-d-1-thiogalactopyranoside (IPTG) and grown overnight at 18 °C.

wt Tau was purified using a similar procedure described by Barghorn et al. [43]. Briefly, wt Tau cell pellets were resuspended in 50 mM NaCl, 5 mM DTT, 50 mM sodium phosphate, pH 6.5, and supplemented with a protease inhibitor cocktail (GenDEPOT, Barker, TX, USA). The cells were lysed using a homogenizer (Avestin, Ottawa, ON, Canada). Additional salt was then added (for a final concentration of 450 mM NaCl) before the solution was incubated for 20 min in hot water (~80–90 °C). The supernatant was concentrated, diluted to a final salt concentration of 50 mM NaCl, and purified by FPLC (Bio-Rad, Hercules, CA, USA) using a salt gradient applied to a heparin sepharose HP column (GE, Marlborough, MA, USA). Fractions containing wt Tau were concentrated and further purified by reverse-phase HPLC (Agilent, Santa Clara, CA, USA), lyophilized, and stored at −80 °C until later use. Purified acetylated Tau (Ac-Tau) was prepared using reverse-phase HPLC after in vitro acetylation of wt Tau (see below).

### 3.2. p300 Histone Acetyltransferase (HAT) Domain Expression and Purification

Enzymatically-active p300 HAT was prepared as previously described [44]. Briefly, p300 HAT and Sir2 expression plasmids (generous gifts from Phillip Cole) were co-transformed into *E. coli* BL21 AI cells (Invitrogen, Carlsbad, CA, USA). Cells were grown at 37 °C in Terrific Broth medium until induction (OD_600_ ≈ 0.8–1.0) with 1 mM IPTG, followed by overnight growth at 18 °C. Both proteins were purified using FPLC (Bio-Rad) with a Talon cobalt resin (GE) and a Q HP sepharose column (GE). Separate p300 HAT and Sir2 fractions were stored in −80 °C until later use. The final storage buffer for p300 HAT is ~150 mM NaCl, 125 mM TCEP, 25% (*v*/*v*) glycerol, 20 mM Tris, pH 8.

### 3.3. In Vitro p300 HAT-Mediated Acetylation Reactions

Acetylation of wt Tau by p300 HAT was performed by combining 500 µL of 86 µM purified wt Tau (dissolved in water), 200 µL of 15 µM p300 HAT, 25 µL of 10 mM acetyl-CoA (Sigma, Saint Louis, MO, USA), and 25 µL of 1 M Tris, pH 8. The acetylation reaction was allowed to proceed for three days at RT (unless stated otherwise).

### 3.4. Western Blot of Acetylated Tau

Tau acetylation was verified by western blot against acetyl-lysine. 100-ng samples of Ac-Tau, wt Tau and BSA were loaded on a 4–20% gradient SDS-PAGE gel (Mini-PROTEAN TGX Precast Gels, Bio-Rad). After electrophoresis, the gel was transferred to a polyvinylidene fluoride (PVDF) membrane using the Trans-Blot Turbo Transfer System, following the manufacturer’s protocols (Bio-Rad). After incubation with 5% (*w*/*v*) nonfat milk in TBS-T (150 mM NaCl, 0.1% Tween-20, 20 mM Tris-HCl, pH 7.5) at RT for 2 h, the membrane was incubated with antibody against acetyl-lysine (1:100 in 1% nonfat milk/TBS-T; sc-32268, Santa Cruz Biotech, Dallas, TX, USA) overnight at 4 °C. The membrane was washed six times for 10 min with TBS-T and incubated with HRP-conjugated anti-mouse antibody (1:1000; #7076, Cell Signaling, Danvers, MA, USA) at RT for 30 min. The membrane was washed six times and developed with Clarity Western ECL Substrate according to the manufacturer’s protocols (Bio-Rad). Chemiluminescent signals were measured using ChemiDoc MP Image System (Bio-Rad).

### 3.5. Mass Spectrometry of Acetylated Tau

Ac-Tau sample was boiled in 30 µL of 1× NuPAGE LDS sample buffer (Invitrogen) and subjected to SDS-PAGE (NuPAGE 10% Bis-Tris gel, Invitrogen) then visualized with Coomassie Brilliant blue-stain. The SDS-PAGE gel containing the band corresponding to Tau was excised, destained, and subjected to in-gel digestion using 100 ng trypsin (#T9600, GenDepot). The digested peptides were resuspended in 10 µL of 0.1% formic acid and subjected to a nanoHPLC-MS/MS system with an EASY-nLC 1200 coupled to Fusion Tribrid Orbitrap Lumos mass spectrometer (Thermo Fisher, Waltham, MA, USA). The peptides were loaded onto a Reprosil-Pur Basic C18 (1.9 µm, Dr. Maisch GmbH, Ammerbuch-Entringen, Germany) pre-column of 2 cm × 100 µm size. The pre-column was switched in-line with an in-housed 50 mm × 150 µm analytical column packed with Reprosil-Pur Basic C18 equilibrated in 0.1% formic acid. The peptides were eluted using a 45-min discontinuous gradient of 4–28% acetonitrile/0.1% formic acid at a flow rate of 750 nL/min. The eluted peptides were directly electro-sprayed into mass spectrometer operated in the data-dependent acquisition mode acquiring fragmentation spectra of the top 30 strongest ions under direct control of Xcalibur software (4.0; Thermo Fisher). Parent MS spectrum was acquired in the Orbitrap with full MS range of 300–1400 *m*/*z* in the resolution of 120,000. CID fragmented MS/MS spectrum was acquired in ion-trap with rapid scan mode. Obtained MS/MS spectra were searched against the target-decoy human refseq database (June 2015 release, containing 73,637 entries) in Proteome Discoverer 1.4 interface (Thermo Fisher) with the Mascot algorithm (Mascot 2.4, Matrix Science, London, UK). Variable modifications of lysine and arginine acetylation, methionine oxidation, and N-terminal acetylation were allowed. The precursor mass tolerance was confined within 20 ppm with fragment mass tolerance of 0.5 Da and with a maximum of two missed cleavages allowed. The peptides identified in the Mascot results file were validated with a 5% false discover rate (FDR) and subjected to manual verification to confirm lysine acetylation.

### 3.6. Microscopy Imaging

Liquid-liquid phase separation (LLPS) experiments were performed using variable protein (wt Tau and Ac-Tau; 2.5–20 µM) and salt (0–250 mM NaCl) concentrations in 5 mM sodium phosphate buffer, pH 7.8. 10–20-µL drops were pipetted onto 35-mm glass bottom dishes (ibidi, Martinsried, Germany) and immediately monitored for droplet formation (with incubation time of 5 min). For heparin-induced LLPS experiments, heparin (8–25 kDa; Santa Cruz Biotech) was mixed with wt Tau or Ac-Tau at approximately 1:4 molar ratio (heparin:protein). LLPS experiments in the presence of crowding agent were carried out using 30 µM wt Tau or Ac-Tau in 10% PEG-8K, αβγ buffer (10 mM glycine, 10 mM acetate, 10 mM sodium phosphate, pH 7.5). Microscopy images were recorded at RT using an FL EVOS imaging system (Invitrogen).

### 3.7. Thioflavin T (Th T) Aggregation Assay

Heparin-induced wt Tau and Ac-Tau aggregation were detected following changes in Th T fluorescence using a Biotek Synergy H1 plate reader, employing 440 nm excitation and 480 nm emission wavelengths. Protein aggregation reactions were conducted using 20 µM wt Tau or Ac-Tau in 5 µM heparin, 10 µM Th T (GenDepot), 0.25 mM TCEP, 5 mM sodium phosphate, pH 7.8. Aggregation kinetics were monitored for ~24 h.

### 3.8. Microtubule Assembly Assay

The ability of wt Tau and Ac-Tau to promote microtubule assembly was investigated using fluorescence imaging. Rhodamine-labeled and unlabeled tubulin heterodimers (1:20 ratio; Cytoskeleton, Inc., Denver, CO, USA) were mixed with wt Tau or Ac-Tau (9 µM tubulin heterodimers and 27.5 µM Tau) in a final buffer condition of 0.2 mM MgCl_2_, 0.1 mM GTP, 50 µM EDTA, 9 mM sodium PIPES, pH 6.9. Microtubule formation was visually monitored using an FL EVOS fluorescence microscope (Invitrogen) starting from 10 min up to 18 h.

## Abbreviations

Ac-Tau	Hyperacetylated Tau
AD	Alzheimer’s disease
IDP	Intrinsically disordered protein
HAT	Histone acetyltransferase
LLPS	Liquid-liquid phase separation
PTMs	Post-translational modifications
wt	Wild-type
